# Goalie: Defending Against Correlated Value and Sign Encoding Attacks

**DOI:** 10.3390/e27030323

**Published:** 2025-03-20

**Authors:** Rongfei Zhuang, Ximing Fu, Chuanyi Liu, Peiyi Han, Shaoming Duan

**Affiliations:** 1 School of Computer Science and Technology, Harbin Institute of Technology, Shenzhen 518055, China; 2 Peng Cheng Laboratory, Shenzhen 518055, China

**Keywords:** privacy preserving, machine learning security, malicious model detection, data sharing, model parameter distribution

## Abstract

In this paper, we propose a method, namely Goalie, to defend against the correlated value and sign encoding attacks used to steal shared data from data trusts. Existing methods prevent these attacks by perturbing model parameters, gradients, or training data while significantly degrading model performance. To guarantee the performance of the benign models, Goalie detects the malicious models and stops their training. The key insight of detection is that encoding additional information in model parameters through regularization terms changes the parameter distributions. Our theoretical analysis suggests that the regularization terms lead to the differences in parameter distributions between benign and malicious models. According to the analysis, Goalie extracts features from the parameters in the early training epochs of the models and uses these features to detect malicious models. The experimental results show the high effectiveness and efficiency of Goalie. The accuracy of Goalie in detecting the models with one regularization term is more than 0.9, and Goalie has high performance in some extreme situations. Meanwhile, Goalie takes only 1.1 ms to detect a model using the features extracted from the first 30 training epochs.

## 1. Introduction

One of the challenges in developing Artificial Intelligence (AI) applications is the scarcity of varied, high-quality data. However, many regulatory restrictions and privacy issues pose barriers to sharing data. A novel solution to this problem is sharing data in data trusts [1,2]. Data providers can safely share data in data trusts to obtain benefits. Data users can use the shared data to train machine learning (ML) models in a workspace provided by data trusts and take out the trained ML models rather than the original data.

As stated in [3], when the data holders use the malicious analysis algorithms provided by third parties to train ML models on their data, the data holders’ data will be leaked through the trained ML models. Similarly, malicious data users may launch some attacks mentioned in [3] to steal the data shared in the data trust. For example, malicious data users can steal the data by LSB encoding attack, capacity abuse attack, correlated value encoding attack, or sign encoding attack. More detailed descriptions of these attacks are given in Section 2.2. We find that the LSB encoding and capacity abuse attacks are relatively easy to defend against. However, the correlated value and sign encoding attacks are more insidious and difficult to defend against. Since the attacker can accurately recover mass training data through correlated value encoding and sign encoding attacks, these attacks are more threatening. For example, the attacker can reconstruct 200 complete images from the FaceScrub dataset, 455 complete images from the CIFAR-10 dataset [4], and 300 complete images from the LFW dataset [3]. The data owner must have a priori knowledge of ’normal’ models to detect model anomalies in correlated value and sign encoding attacks [3]. This kind of anomaly detection is challenging for humans. Therefore, this paper focuses on the defense of correlated value and sign encoding attacks.

Several methods have been proposed to protect the training data. Abadi et al. [5] perturbed gradients to reduce the information related to the training data in the parameters. Golatkar, Achille, and Soatto [6] added noise to model parameters to make models forget specific data. However, these methods perturb the gradients or parameters of the model regardless of whether the model is benign or malicious. This will cause degradation in the performance of the benign model. For example, the model accuracy can decrease by 0.13 [5] and 0.108 [6] when perturbing gradients and parameters. In addition, several studies perturb or encrypt training data to protect privacy [7,8,9,10]. Even if the attackers steal the training data that have been perturbed or encrypted, they cannot recover these data. However, adding perturbations to data or encrypting data can degrade the model’s accuracy [11,12,13] and incur heavy computation overhead [14].

Existing methods prevent the models from encoding training data by adding noise to the models. However, these methods degrade the performance of the benign models. This paper adopts a different approach that detects and stops the malicious models before they fully encode the training data.

In summary, our key contributions are as follows:

- We propose Goalie, which defends against correlated value and sign encoding attacks by detecting malicious models and stopping their training. Goalie extracts several parameter features from the model in the early epoch of model training to distinguish between benign and malicious models. If Goalie detects that a model is malicious, Goalie can stop training the model to prevent it from encoding training data. Since Goalie only restricts the malicious models, Goalie can avoid affecting the performance of the benign models.
- We theoretically analyze different regularization terms used in the benign and malicious models. Based on the analysis, we discover that there are four significantly different parameter distributions when four different regularization terms, including two benign and two malicious regularization terms, are used separately in the model. The analysis demonstrates that the features extracted by Goalie based on the discrepancy of the parameter distributions can distinguish between benign and malicious models.
- Our experimental results show that Goalie can accurately detect benign and malicious models trained on the different datasets with an accuracy of more than 0.9. Even in the extreme situation where the parameter distributions of benign and malicious models are similar, Goalie can still achieve an accuracy above 0.9. If the attacker uses both benign and malicious regularization terms in the model at the same time, Goalie can also detect malicious models with a $F_{1}$ score greater than 0.9. Meanwhile, Goalie takes only about 1.1 ms to detect a model using the parameter features extracted from the first 30 training epochs, which is highly efficient.

The rest of this paper is organized as follows. Section 2 provides background knowledge about the data trusts and data stealing attacks. Section 3 presents the abilities and objectives of the attacker and defender. Section 4 introduces our key insight and the method used to detect the malicious models. Section 5 analyzes the parameter distributions of the models with different regularization terms. Section 6 presents our experiments and results. Section 7 describes related work on the defense and attack methods. Section 8 concludes this paper.

## 2. Background

In this section, we describe some background knowledge. We illustrate data trusts and introduce some attacks in the data trusts.

### 2.1. Data Trusts

**Figure 1:**
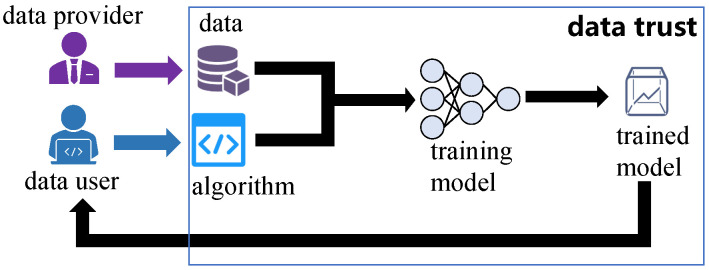
Data trusts.

As shown in Figure 1, data trusts are virtual places where data are made available to share. The data providers have a lot of valuable data D and desire to share D with others to receive benefits. The data providers share D through the data trusts. The data trusts manage the data shared by data providers and ensure that unauthorized data users cannot access these data. Meanwhile, the data trusts can access the data shared by the data providers. The data users wish to use data via the data trusts. In the data trusts, the data users can use data D by submitting training algorithms to the data trusts to train ML models on the data D. The data users cannot directly obtain data D from the data trust. The data users can only obtain the trained ML models from the data trusts. The ML model is a function $f_{w}:D\mapsto y$, where w are the model parameters and y are the prediction results of the model.

### 2.2. Attacks

The attacker can launch several attacks mentioned in [3] to steal training data from the data trusts.

#### 2.2.1. LSB Encoding Attack

In this attack, the attacker maps the training data D to a set of bit strings to obtain secret values s. The attacker trains a benign model and sets the lower *b* bits of each parameter in this model to a *b*-length bit string of s. After obtaining the trained model, the attacker can read the lower *b* bits of each parameter in this model to obtain the secret values s and transform s into the training data D.

As described in [3], the LSB encoding attack can be defended by replacing the lower *b* bits of the model parameters with random noise. Since the lower bits of model parameters essentially do not matter for model accuracy, replacing these bits will destroy any information potentially encoded in these bits without any impact on the model’s performance.

#### 2.2.2. Capacity Abuse Attack

In this attack, the attacker maps each data point of the training data D to a set of float numbers to obtain secret values s. The attacker can generate a set of malicious data in the data trusts and let the labels of malicious data encode s. However, the attacker can not obtain these data and labels from the data trusts. The attacker uses these malicious data as the augmented dataset to train the model together with the training dataset. When the model is trained well, it can accurately predict the labels of malicious data. After obtaining the trained model, the attacker uses the same method to generate the malicious data and obtains their labels through the obtained model. Then, the attacker decodes these labels to obtain the secret values s and maps s to the training data D.

Since the capacity abuse attack does not require the attacker to have any prior knowledge about the training data [3], the malicious data generated by the attacker will be different from the training data. It is easy to identify the capacity abuse attack by distinguishing the malicious data from the training data in this attack.

#### 2.2.3. Correlated Value Encoding Attack

In the correlated value encoding attack (COR), the attacker maps each data point of the training data D to a set of float numbers to obtain secret values s. Then, the attacker uses the correlated value encoding regularization term $\Omega_{COR}$ as an extra term in the objective function to map the secret values s to the model parameters w, where

(1) $$\Omega_{COR}\left(w,s\right)=-\lambda_{c}\frac{|\sum_{i=1}^{l}\left(w_{i}-\bar{w}\right)\left(s_{i}-\bar{s}\right)|}{\sqrt{\sum_{i=1}^{l}{\left(w_{i}-\bar{w}\right)}^{2}}\sqrt{\sum_{i=1}^{l}{\left(s_{i}-\bar{s}\right)}^{2}}}.$$

In Equation (1), the secret values $s\in R^{l}$, *l* is the number of parameters, $\bar{w}$ and $\bar{s}$ are the mean values of w and s, and $\lambda_{c}\in \left[0,\infty \right)$ is the coefficient of the regularization term. This regularization term $\Omega_{COR}$ is the negative absolute value of the Pearson correlation coefficient of w and s. As $\Omega_{COR}$ becomes smaller, the correlation between model parameters w and secret values s gradually increases. During the model training process, $\Omega_{COR}$ drives the model’s objective function towards a local minimum where the secret values and the parameters are highly correlated. After obtaining the trained model, the attacker can easily map the trained model parameters w to the secret values s because correlated parameters are approximately linear transformations of the secret values [3]. Then, the attacker can map s to the training data D.

#### 2.2.4. Sign Encoding Attack

In the sign encoding attack (SGN), the attacker maps 1 and 0 in the binary representation of training data D to 1 and −1 to obtain secret values s. Then, the attacker uses the sign encoding regularization term $\Omega_{SGN}$ as an extra term in the objective function to map the secret values s to the model parameters w, where

(2) $$\Omega_{SGN}\left(w,s\right)=\frac{\lambda_{s}}{l}\left|\sum_{i=1}^{l}\max\left(0,-w_{i}s_{i}\right)\right|.$$

In Equation (2), the secret values $s\;\in {\left\{-1,1\right\}}^{l}$, *l* is the number of parameters, and $\lambda_{s}\in \left[0,\infty \right)$ is the regularization term coefficient. When $w_{i}$ and $s_{i}$ have the same sign during the model training process, zero penalty is added to the model’s objective function by $\Omega_{SGN}$. When $w_{i}$ and $s_{i}$ have different signs during the model training process, a $|w_{i}s_{i}|$ penalty is added to the model’s objective function by $\Omega_{SGN}$. Therefore, $\Omega_{SGN}$ constrains the sign of the parameter w to be the same as the sign of the secret values s. During the model training process, $\Omega_{SGN}$ drives the model’s objective function towards a local minimum where almost sign constraints of the parameters are met. After obtaining the trained model, the attacker can read the signs of the trained model parameters w and then interpret them as bits of the training data D [3].

### 2.3. Limitation of Current Defense Techniques

Current techniques add noise to the data, model gradients, or model parameters to defend against correlated value and sign encoding attacks. They significantly reduce the performance of the model. We illustrate this with an experimental example. When the sign encoding attack is applied to a residual network [15] (ResNet) trained on CIFAR-10, the training data can be recovered with a Mean Absolute Error (MAE) of 2.88. At the same time, the ResNet has an accuracy of 0.91. Adding Gaussian noise with a mean of 0 and a standard deviation of 0.05 to the ResNet parameters increases the MAE to 24.38 and reduces the accuracy of the ResNet to 0.74. It reduces the quality of the recovered data at a cost of 0.17 model accuracy.

## 3. Threat Model

In this section, we present our threat model. We illustrate the abilities and objectives of the attacker and defender in data trusts.

### 3.1. Attacker

We assume that the attacker is a malicious data user in the data trusts. We assume that the attacker is not allowed to access data D directly from the data trusts. We assume that the attacker can use the data D by submitting an algorithm to the data trusts to train an ML model $f_{w}$. To steal data from the data trusts, the attacker can add a malicious regularization term to the algorithm [3] before they submit this algorithm to the data trusts. We assume that the attacker cannot observe the data D or the execution of the model training algorithm during the model training process. We also assume that the attacker can obtain the trained model $f_{w}$ after the model training process is completed. Based on these assumptions, the attacker can launch the correlated value and sign encoding attacks described in Section 2.2 to steal the data D.

**Attacker’s Objectives:** The attacker’s main objective is to obtain the training data D through the parameters w of a trained model $f_{w}$. The attacker encodes the information of training data D in the parameters w of model $f_{w}$ during the model training process. After the attacker obtains the trained model $f_{w}$, the attacker decodes the training data D from the model parameters w.

### 3.2. Defender

We assume that the data trusts act as defenders, and the data trusts are secure. We assume that the defender can obtain the parameters w of the model $f_{w}$ after each training epoch. We assume that the defender can access data D shared on the data trusts. We also assume that the defender has no prior knowledge of the training algorithm used by the data user.

**Defender’s Objectives:** The defender’s objective is to efficiently and accurately distinguish malicious models from benign models. The defender treats the model with the $l_{1}$-norm regularization term ($l_{1}$-model), the model with the $l_{2}$-norm regularization term ($l_{2}$-model), and the model without regularization terms (no-model) as benign models. The defender treats the model with the correlated value encoding regularization term (COR-model) and the model with the sign encoding regularization term (SGN-model) as malicious models. To prevent attackers from stealing data as early as possible, the defender should detect the malicious models during the early epoch of model training. The defender uses a set of parameters $\left\{w_{i}|i\in \left[1,k\right]\right\}$ that are obtained from the first *k* training epochs of a model $f_{w}$ trained on the data trusts to determine if this model is malicious.

## 4. Design of Goalie

In this section, we propose a method, named Goalie, to detect and stop the malicious models in the correlated value and sign encoding attacks. First, an overview of Goalie is discussed. Then, the key insight of malicious model detection is outlined. Finally, the details of the two stages in Goalie, i.e., feature extraction and judgment, are presented.

### 4.1. Overview

**Figure 2:**
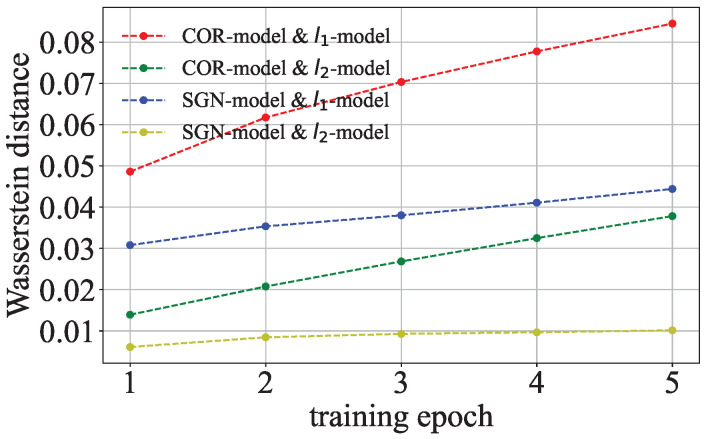
Wasserstein distance between the parameter distributions of the models with different regularization terms.

Our key insight is that the parameter distributions of malicious models differ from those of benign models when the attackers encode training data into the parameters of the malicious models through regularization terms. The authors of [3] show that the parameter distribution of a trained benign model is different from that of a trained malicious model. Figure 2 shows the Wasserstein distance [16] between the parameter distributions of the benign and malicious models trained on CIFAR-10 by ResNet with different regularization terms. The Wasserstein distances between the different parameter distributions increase as the training epoch increases. The data trusts try to detect malicious models as early as possible. However, the differences in the parameter distributions between benign and malicious models are not obvious in the early epochs of model training. It is difficult to detect malicious models based on the parameter distributions at this stage. The regularization terms gradually change the parameter distributions over several training epochs. It is possible to distinguish the models with different regularization terms based on the evolution patterns of the parameter distributions during the early epoch of model training. We find two discrepancy phenomena between different parameter distributions to distinguish the benign and malicious models.

**Figure 3:**
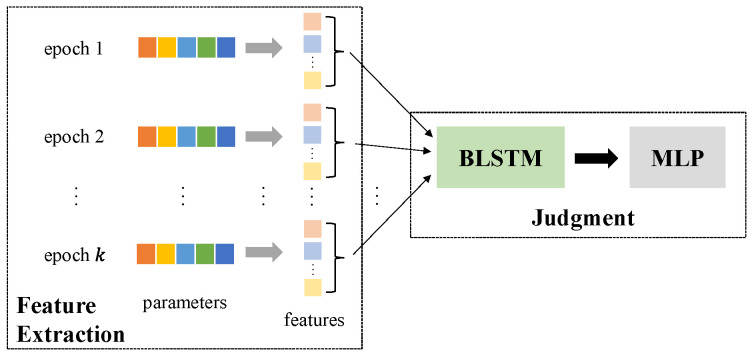
Goalie architecture.

We propose Goalie to defend against correlated value and sign encoding attacks by detecting and stopping the malicious models that encode training data. Since stopping the training of malicious models is a simple task, we focus on the detection of malicious models in this paper. The architecture of Goalie is depicted in Figure 3. Goalie is composed of two stages, namely *feature extraction* and *judgment*. In the feature extraction stage, Goalie collects the parameters of a training model in each training epoch during the early stage of model training and extracts some features from these parameters based on the discrepancy phenomena we discovered. In the judgment stage, Goalie uses a machine learning method to determine whether this training model is malicious based on these features. Typically, the input dimension of a machine learning method is fixed, while the number of parameters in different models varies considerably. In addition, the number of parameters in certain models is too large [17] to increase the overhead of the machine learning method or to make the machine learning method unusable. As a result, we do not directly use the parameters of the training model to detect malicious models. Instead, we detect malicious models based on the features extracted from the parameters of the training model.

### 4.2. Feature Extraction

The different regularization terms used in benign and malicious models lead to differences in their parameter distributions. We discover two discrepancy phenomena between the parameter distributions of benign and malicious models by comparing the parameter distributions of ResNet models trained on the CIFAR-10 dataset. In the feature extraction stage, Goalie extracts several features from the model parameters in each training epoch based on these phenomena.

#### 4.2.1. Phenomenon 1: Difference in the Dispersion of the Distributions

**Figure 4:**
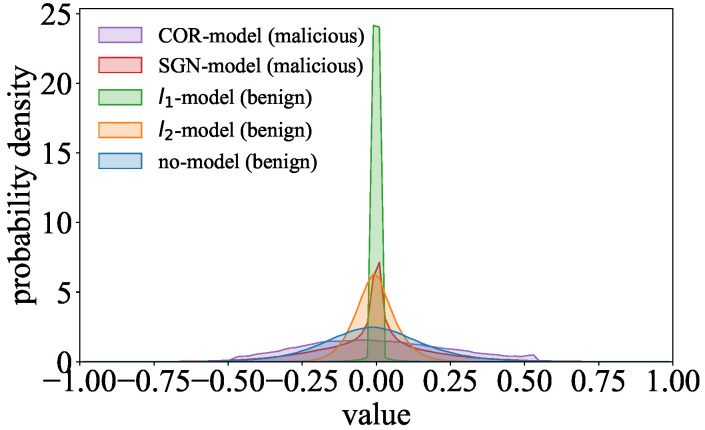
Comparison of the parameter distributions in malicious and benign models.

We find that the data in the parameter distribution of the malicious models are more scattered than those in the parameter distributions of the benign models. As shown in Figure 4, the parameter distribution of the COR-model is more stretched than those of the benign models. Similarly, the parameter distribution of the SGN-model is significantly more stretched than those of the benign models in the regions away from the center.

- Goalie extracts the standard deviation σ as the feature to represent the distribution’s dispersion. For example, σ is about 0.21 for the COR-model and about 0.09 for the $l_{2}$-model in the 30th training epoch of ResNet on CIFAR-10. When σ becomes larger, the data in the distribution will be more scattered.
- Goalie extracts the inter-percentile range $P_{b}-P_{a}$ as the feature to represent the local dispersion of the distribution, where $P_{a}$ and $P_{b}$, respectively, denote the *a*-th percentile and *b*-th percentile in the distribution. For example, $P_{16}-P_{2}$ is about 1.45 for the SGN-model and about 1.10 for the $l_{2}$-model in the 30th training epoch of ResNet on CIFAR-10. If $P_{b}-P_{a}$ in the distribution is larger, the data will be more scattered in the interval $\left[P_{b},P_{a}\right]$. Since different models’ parameters may be on different scales, we standardize the parameters by z-score [18] before calculating the inter- percentile range.

#### 4.2.2. Phenomenon 2: Differences in the Data Proportion of the Distributions

We find that the data proportion in some regions of the parameter distributions for the malicious and benign models is different. We split the distribution into five parts: a head region, two middle regions, and two tail regions. According to Chebyshev’s inequality, the intervals [μ−2σ,μ+2σ] and [μ−3σ,μ+3σ], respectively, contain at least 75% of the data and 88.8% of the data in a distribution [19]. We consider the interval [μ−2σ,μ+2σ] as the distribution’s head region, the intervals [μ−3σ,μ−2σ] and [μ+2σ,μ+3σ] as the distribution’s middle regions, and the intervals (−∞,μ−3σ] and [μ+3σ,+∞) as the distribution’s tail regions. As shown in Figure 4, the data proportion of the parameter distribution in the head region is relatively similar for the SGN-model and benign models. For example, the proportion $p_{\left[\mu -2\sigma ,\mu +2\sigma \right]}$ is about 0.941 for the SGN-model and about 0.943 for the $l_{1}$-model in the 30th training epoch of ResNet on CIFAR-10. However, the probability densities of the SGN-model’s parameter distribution are higher in the middle regions than those of the benign models’ parameter distributions in Figure 4. Moreover, we can observe that the probability density of the COR-model’s parameter distribution is reduced to 0 when the data value is about −0.5 or 0.5, whereas the probability densities of the benign models’ parameter distributions gradually close to 0 in the tail regions.

- Goalie extracts the proportion $p_{\left[\mu -3\sigma ,\mu -2\sigma \right]}$ and $p_{\left[\mu +2\sigma ,\mu +3\sigma \right]}$ as the features to represent the data proportion of the distribution in the middle regions. For example, $p_{\left[\mu -3\sigma ,\mu -2\sigma \right]}$ is about 0.022 for the SGN-model and about 0.015 for the $l_{2}$-model in the 30th training epoch of ResNet on CIFAR-10.
- Goalie extracts the proportions $p_{\left(-\infty ,\mu -3\sigma \right]}$ and $p_{\left[\mu +3\sigma ,+\infty \right)}$ as the features to represent the data proportion of the distribution in the tail regions. For example, $p_{\left(-\infty ,\mu -3\sigma \right]}$ is about 0.0002 for the COR-model and about 0.0084 for the $l_{1}$-model in the 30th training epoch of ResNet on CIFAR-10.

The features Goalie extracted from the model parameters based on these two discrepancy phenomena are shown in Table 1.

### 4.3. Judgment

As shown in Figure 3, Goalie uses a bidirectional long short-term memory (BLSTM) network and a multilayer perceptron (MLP) to judge whether a model is malicious or not in the judgment stage. Goalie first uses BLSTM to process the *k*-length feature sequences T extracted from the model parameters in the first *k* training epochs. The *i*-th element of T is a vector composed of the features in Table 1 extracted from the model parameters in the *i*-th training epoch. Then, Goalie uses an MLP to classify the output of BLSTM into two categories: benign and malicious models.

BLSTM takes the feature sequence T as input. T is read sequentially by BLSTM from both directions. The evolution information of the parameter features is modeled by employing temporal recurrence in BLSTM. The MLP takes the output vector of BLSTM as input. The output layer of the MLP has two neurons and is used to perform binary classification. The MLP outputs a vector $\left(p_{0},p_{1}\right)$, where $p_{0}$ and $p_{1}$ correspond to the probabilities of benign and malicious models, respectively. For $p_{0}>p_{1}$, Goalie judges the model as benign. Otherwise, Goalie judges the model as malicious.

## 5. Analysis

In this section, we theoretically analyze the parameter distributions for models with different regularization terms. Meanwhile, we prove that regularization terms make parameter distributions different.

The objective function $\tilde{J}\left(w,D,y\right)$ of the model has two components. One component is the standard objective function J(w,D,y), which enables the model to complete the given target task. The other component is the regularization term Ω, which prevents the model from overfitting or makes the model parameters encode the training data. The objective function of a model can be presented as

(3) $$\underset{w}{\mathrm{argmin}}\tilde{J}\left(w,D,y\right)=\underset{w}{\mathrm{argmin}}\left[J\left(w,D,y\right)+\lambda \Omega \right],$$

where λ∈[0,∞) is the regularization term’s coefficient.

Both J(w,D,y) and Ω affect the model parameters w during the model training process. If J(w,D,y) can completely counteract the effect of Ω on the model, then Ω cannot prevent the model from overfitting or cannot make the model parameters encode the training data. In this case, the attacker cannot steal the training data. If the attacker can successfully launch correlated value and sign encoding attacks, then the regularization term will undoubtedly affect the model parameters during the model training process.

### 5.1. Parameter Distribution of Model with $l_{1}$-Norm Regularization Term

The $l_{1}$-norm regularization term of model parameters w is

(4) $$\Omega_{l_{1}}\left(w\right)={∥w∥}_{1}=\sum_{i}\left|w_{i}\right|.$$

Accordingly, the objective function of the $l_{1}$-model can be presented as

(5) $$\underset{w}{\mathrm{argmin}}\tilde{J}\left(w,D,y\right)=\underset{w}{\mathrm{argmin}}[J\left(w,D,y\right)+\lambda \sum_{i}|w_{i}\left|\right].$$

**Figure 5:**
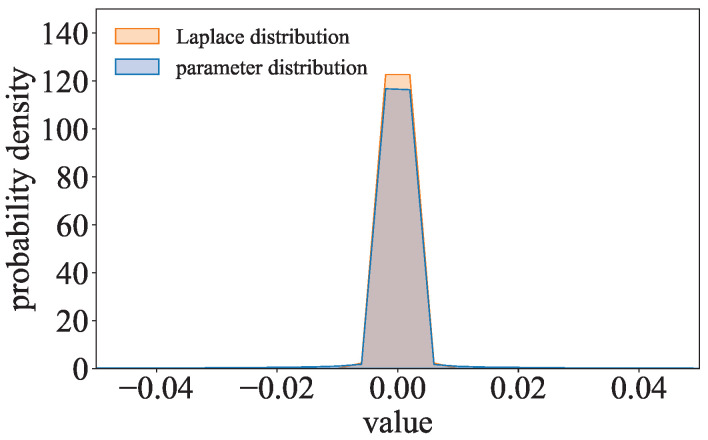
Comparison of the Laplace distribution and the parameter distribution of the $l_{1}$-model.

We can impose the following transformation on Equation (5)

(6) $$\begin{array}{cc} \; & \underset{w}{\mathrm{argmin}}\tilde{J}\left(w,D,y\right)=\underset{w}{\mathrm{argmin}}[J\left(w,D,y\right)+\lambda \sum_{i}|w_{i}\left|-\sum_{i}\log\frac{\lambda }{2}+\sum_{i}\log\frac{\lambda }{2}\right]\\ = & \underset{w}{\mathrm{argmin}}[J\left(w,D,y\right)-(\sum_{i}\log\exp(-\lambda |w_{i}\left|\right)+\sum_{i}\log\frac{\lambda }{2})+\sum_{i}\log\frac{\lambda }{2}]\\ = & \underset{w}{\mathrm{argmin}}[J\left(w,D,y\right)-\log\prod_{i}[\frac{\lambda }{2}\exp(-\lambda |w_{i}\left|)\right]+\sum_{i}\log\frac{\lambda }{2}]\\ = & \underset{w}{\mathrm{argmin}}\left[J\left(w,D,y\right)-\log\prod_{i}p\left(w_{i}\right)+\sum_{i}\log\frac{\lambda }{2}\right], \end{array}$$

where $p\left(w_{i}\right)=\lambda \exp(-\lambda |w_{i}\left|\right)/2$. Inspired by $\lambda \exp(-\lambda |w_{i}|)/2$, the parameter distribution of the $l_{1}$-model is compared to the Laplace distribution. As shown in Figure 5, the parameter distribution of ResNet with the $l_{1}$-norm regularization term trained on CIFAR-10 is close to the Laplace distribution. The parameters of the $l_{1}$-model can be considered to follow a Laplace-like distribution.

### 5.2. Parameter Distribution of Model with $l_{2}$-Norm Regularization Term

The $l_{2}$-norm regularization term of model parameters w is

(7) $$\Omega_{l_{2}}\left(w\right)={∥w∥}_{2}^{2}=\sum_{i}w_{i}^{2}.$$

Accordingly, the objective function of the $l_{2}$-model can be presented as

(8) $$\underset{w}{\mathrm{argmin}}\tilde{J}\left(w,D,y\right)=\underset{w}{\arg\min}\left[J\left(w,D,y\right)+\lambda \sum_{i}w_{i}^{2}\right].$$

**Figure 6:**
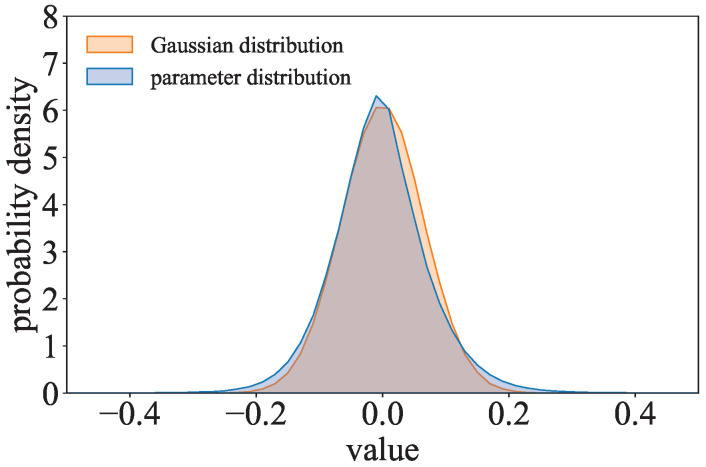
Comparison of the Gaussian distribution and the parameter distribution of the $l_{2}$-model.

We can impose the following transformation on Equation (8)

(9) $$\begin{array}{cc} \; & \underset{w}{\mathrm{argmin}}\tilde{J}\left(w,D,y\right)=\underset{w}{\mathrm{argmin}}\left[J\left(w,D,y\right)+\lambda \sum_{i}w_{i}^{2}-\sum_{i}\log\sqrt{\frac{\lambda }{\pi }}+\sum_{i}\log\sqrt{\frac{\lambda }{\pi }}\right]\\ = & \underset{w}{\mathrm{argmin}}\left[J\left(w,D,y\right)-\left(\sum_{i}\log\exp\left(-\lambda w_{i}^{2}\right)+\sum_{i}\log\sqrt{\frac{\lambda }{\pi }}\right)+\sum_{i}\log\sqrt{\frac{\lambda }{\pi }}\right]\\ = & \underset{w}{\mathrm{argmin}}\left[J\left(w,D,y\right)-\log\prod_{i}\left[\sqrt{\frac{\lambda }{\pi }}\exp\left(-\lambda w_{i}^{2}\right)\right]+\sum_{i}\log\sqrt{\frac{\lambda }{\pi }}\right]\\ = & \underset{w}{\mathrm{argmin}}\left[J\left(w,D,y\right)-\log\prod_{i}p\left(w_{i}\right)+\sum_{i}\log\sqrt{\frac{\lambda }{\pi }}\right], \end{array}$$

where $p\left(w_{i}\right)={\left(\lambda /\pi \right)}^{1/2}\exp\left(-\lambda w_{i}^{2}\right)$. Inspired by ${\left(\lambda /\pi \right)}^{1/2}\exp\left(-\lambda w_{i}^{2}\right)$, the parameter distribution of the $l_{2}$-model is compared to the Gaussian distribution. As shown in Figure 6, the parameter distribution of ResNet with the $l_{2}$-norm regularization term trained on CIFAR-10 is close to the Gaussian distribution. The parameters of the $l_{2}$-model can be considered to follow a Gaussian-like distribution.

### 5.3. Parameter Distribution of Model with Correlated Value Encoding Regularization Term

The correlated value encoding regularization term $\Omega_{COR}$ is given in Equation (1). The objective function of the COR-model can be presented as

(10) $$\begin{array}{cc} \; & \underset{w}{\mathrm{argmin}}\tilde{J}\left(w,D,y\right)=\underset{w}{\mathrm{argmin}}\left[J\left(w,D,y\right)-\lambda_{c}\frac{|\sum_{i=1}^{l}\left(w_{i}-\bar{w}\right)\left(s_{i}-\bar{s}\right)|}{\sqrt{\sum_{i=1}^{l}{\left(w_{i}-\bar{w}\right)}^{2}}\sqrt{\sum_{i=1}^{l}{\left(s_{i}-\bar{s}\right)}^{2}}}\right]. \end{array}$$

We find that $\Omega_{COR}$ is the negative absolute value of the Pearson correlation coefficient, which measures the correlation between model parameters w and the secret values s. Substituting $w_{i}^{*}=w_{i}-\bar{w}$ and $s_{i}^{*}=s_{i}-\bar{s}$ into Equation (10), we can obtain

(11) $$\begin{array}{cc} \; & \underset{w}{\mathrm{argmin}}\tilde{J}\left(w,D,y\right)=\underset{w}{\mathrm{argmin}}\left[J\left(w,D,y\right)-\lambda_{c}\frac{|\sum_{i=1}^{l}w_{i}^{*}s_{i}^{*}|}{\sqrt{\sum_{i=1}^{l}{\left(w_{i}^{*}\right)}^{2}}\sqrt{\sum_{i=1}^{l}{\left(s_{i}^{*}\right)}^{2}}}\right]\\ = & \underset{w}{\mathrm{argmin}}\left[J\left(w,D,y\right)-\lambda_{c}\frac{|w^{*}s^{*}|}{∥w^{*}∥∥s^{*}∥}\right]=\underset{w}{\mathrm{argmin}}[J\left(w,D,y\right)-\lambda_{c}|\cos\theta |], \end{array}$$

where $∥w^{*}∥$ and $∥s^{*}∥$ are the modules of vector $w^{*}$ and vector $s^{*}$, and θ is the angle between vector $w^{*}$ and vector $s^{*}$.

**Figure 7:**
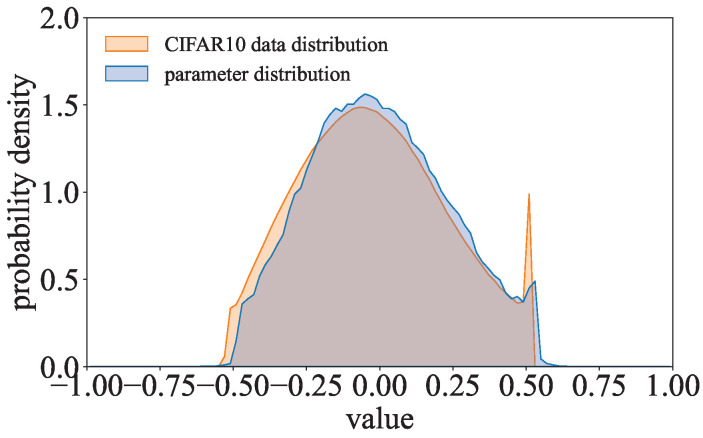
Comparison of the gray-scale value distribution of the CIFAR-10 dataset and the parameter distribution of the COR-model.

When $\Omega_{COR}$ is minimized ideally through training, cosθ of $w^{*}$ and $s^{*}$ is 1 or −1, i.e., $w^{*}$ and $s^{*}$ are parallel. Therefore, ∃λ≠0 and $w^{*}=\lambda s^{*}$. Since $w_{i}^{*}=w_{i}-\bar{w}$ and $s_{i}^{*}=s_{i}-\bar{s}$, we can obtain $w_{i}=\lambda s_{i}+\left(\bar{w}-\lambda \bar{s}\right)$. This means that w are linear transformations of s. We can scale each element of s by λ and add $\bar{w}-\lambda \bar{s}$ to obtain w. Although $\Omega_{COR}$ may not be minimized ideally in practice, the parameters w will also be approximately linear transformations of the secret values s. Since scaling and adding the same constants to each element of the data does not change the type of data distribution, w and s follow the same type of distribution. In other words, $\Omega_{COR}$ can lead to model parameters w and the secret values s having similar distributions. As shown in Figure 7, when the ResNet with the correlated value encoding regularization term is trained on CIFAR-10, the parameter distribution of this model is quite similar to the gray-scale value distribution of the training data.

There are significant differences between distinct data in the training dataset, so the corresponding value representations of these data are different. The data values in the training dataset are so diversified that the data in the value distribution of the training dataset are more scattered. Similar to the training dataset’s value distribution, the data in the parameter distribution of the COR-model will be scattered. Since the domain of the data in the training dataset is finite, the data proportion of the training dataset’s value distribution will drop to 0 directly outside the domain’s boundaries. The data proportion of the COR-model’s parameter distribution will be relatively small as the value distribution of the training dataset is concentrated in the tails.

### 5.4. Parameter Distribution of Model with Sign Encoding Regularization Term

The sign encoding regularization term $\Omega_{SGN}$ is given in Equation (2). The objective function of the SGN-model can be presented as

(12) $$\begin{array}{c} \underset{w}{\mathrm{argmin}}\tilde{J}\left(w,D,y\right)=\underset{w}{\mathrm{argmin}}[J\left(w,D,y\right)+\frac{\lambda_{s}}{l}|\sum_{i=1}^{l}\max\left(0,-w_{i}s_{i}\right)\left|\right]. \end{array}$$

In the general case, there are only some elements in parameters w that have opposite signs to the corresponding elements in secret values s:

(13) $$\max\left(0,-w_{i}s_{i}\right)=\left\{\begin{array}{cc} |w_{i}|, & i\in P\\ 0, & i\in U-P \end{array}\right.,$$

where $U\;=\;\left\{i|i\;\in \;[0,l]\right\}$ is a universal set of parameter subscripts and $P\;=\;\left\{i|i\;\in \;\left[0,l\right],w_{i}s_{i}\;<\;0\right\}$ is a set of parameter subscripts where these parameters’ signs are opposite to the signs of the secret values. Substituting Equation (13) into Equation (12), we have

(14) $$\begin{array}{cc} \; & \underset{w}{\mathrm{argmin}}\tilde{J}\left(w,D,y\right)=\underset{w}{\mathrm{argmin}}[J\left(w,D,y\right)+\frac{\lambda_{s}}{l}|\sum_{i=1}^{l}\max\left(0,-w_{i}s_{i}\right)\left|\right]\\ = & \underset{w}{\mathrm{argmin}}[J\left(w,D,y\right)+\frac{\lambda_{s}}{l}\sum_{i\in P}|w_{i}\left|\right], \end{array}$$

where $P=\left\{i|i\in \left[0,l\right],w_{i}s_{i}<0\right\}$.

**Figure 8:**
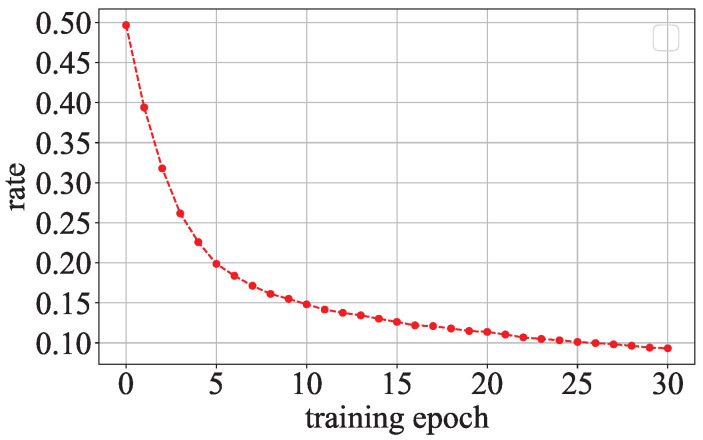
Percentage of SGN-model’s parameters whose signs are different from the signs of the secret values.

**Figure 9:**
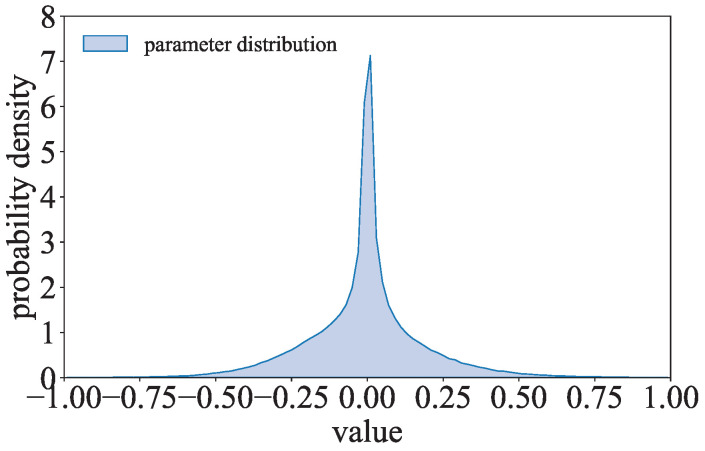
Parameter distribution of the SGN-model.

The regularization term in Equation (14) is similar to the regularization term in Equation (5). $\Omega_{SGN}$ only makes the parameters with opposite signs to the secret values close to 0 during the model training process. Therefore, only some parameters will be close to 0 in the parameter distribution of the model with the sign regularization term. However, $\Omega_{l_{1}}$ makes all parameters close to 0 during the model training process. Therefore, most model parameters will be close to 0 in the parameter distribution of the $l_{1}$-model. As Figure 8 shows, there are only a few parameters whose signs are opposite to the signs of the secret values in each training epoch of a model with the sign encoding regularization term. Furthermore, the percentage of the parameters whose signs are opposite to the signs of the secret values gradually decreases as training epochs increase. As shown in Figure 9, when the ResNet with thesign encoding regularization term is trained on CIFAR-10, the parameter distribution of this model has higher probability densities in the regions away from the center.

In the parameter distribution of the SGN-model, only some data will be close to 0 by the influence of $\Omega_{SGN}$, and the other data may be far from 0. The data proportion of the SGN-model’s parameter distribution will be relatively high in the regions $\left(-\infty ,t_{1}\right)$ and $\left(t_{2},+\infty \right)$ when $t_{1}$ and $t_{2}$ are, respectively, small and large enough. In addition, the parameter distribution of the SGN-model does not have too much data concentrated in the center.

### 5.5. Parameter Distribution of Model Without Regularization Terms

**Figure 10:**
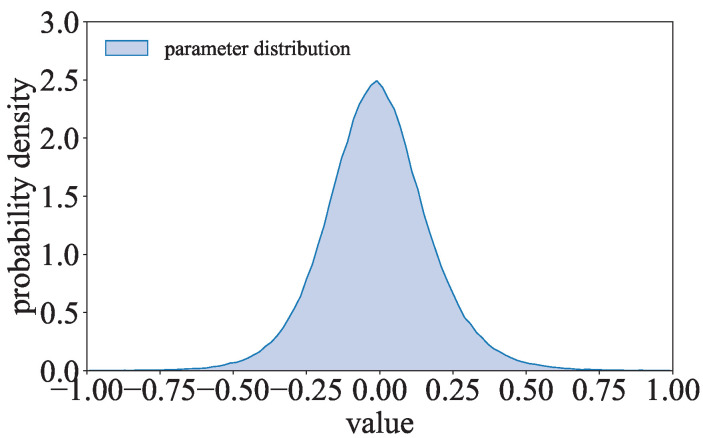
Parameter distribution of the model without any regularization term.

Since the model parameters are updated by minimizing the objective function J(w,D,y) during the model training process, the objective function affects the distribution of the model parameters. However, different machine learning tasks have different objective functions, so we can hardly analyze the parameter distribution through the objective function. We analyze the parameter distribution of the model without any regularization term by observing this parameter distribution. Figure 10 shows the parameter distribution of ResNet without regularization terms trained on CIFAR-10. We can see that most of the parameters in this parameter distribution are concentrated around 0.

### 5.6. Summary and Discussion

According to the above theoretical analysis, we discover that the model parameters follow different distributions when different regularization terms are used for benign and malicious models. With $\Omega_{l_{1}}$, the model’s parameters follow a Laplace-like distribution. With $\Omega_{l_{2}}$, the model’s parameters follow a Gaussian-like distribution. With $\Omega_{COR}$, the model’s parameters approximately follow the value distribution of the training dataset. With $\Omega_{SGN}$, the parameter distribution is different from the Laplace-like and Gaussian-like distributions in the regions $\left(-\infty ,t_{1}\right)$ and $\left(t_{2},+\infty \right)$ when $t_{1}$ and $t_{2}$ are, respectively, small and large enough. When the training dataset follows a Gaussian-like distribution, the parameter distributions of the COR-model and $l_{2}$-model may be indistinguishable. However, the training dataset, in practice, does not completely follow a Gaussian-like distribution due to the finite value domain of the data. Therefore, we can distinguish the models with different regularization terms based on their parameter distributions, which is confirmed by the experiment results in Section 6.2.

We use the regularization term to derive the model parameter distribution. Furthermore, we use the derived distribution to approximate the true parameter distribution. Our method distinguishes malicious models from benign models based on the statistical features extracted from the model parameter distributions. These features are found by comparing the differences in the distributions derived from different regularization terms and are independent of the model architecture. Thus, our method is model-agnostic.

Our method detects the malicious models that encode training data based on the insight that encoding information in model parameters changes the parameter distribution. Therefore, our method is suitable for other attacks that encode training data in model parameters. For example, PCEA [20] uses the regularization term $\lambda \frac{\left||\right|w_{1}+w_{2}+\ldots +w_{k}|-\frac{s}{r}{\left|\right|}_{1}}{l}$ to encode the training data by linearly combining the model parameters. The model parameter w is evenly divided into *k* vectors $w_{i}$, where $w_{i}$ contains the (ik−k+1)th to the ikth parameters. *l* is the length of the stolen private data s, and *r* is the scaling rate. If $|w_{ik-k+1}+w_{ik-k+2}+\ldots +w_{ik}|$ is greater than $\frac{s_{i}}{r}$ and all parameters are non-negative, the regularization term can be written as $\sum_{i}^{l}|w_{ik-k+1}+w_{ik-k+2}+\ldots +w_{ik}|-\sum_{i}^{l}s_{i}={\left||w|\right|}_{1}-\sum_{i}^{l}s_{i}$. At this point, the regularization term is equal to $\Omega_{l_{1}}$. However, the regularization term cannot encode the training data into parameters because w and s are irrelevant. The parameter distributions of malicious and benign models can be distinguished if the regularization term can encode the training data into parameters of the malicious model. Therefore, our method can detect the malicious model if the attack encodes the training data into the parameters.

## 6. Experiments

In this section, we evaluate the performance of Goalie. First, we describe the setup of the experiments. Then, we evaluate the effectiveness and efficiency of Goalie.

### 6.1. Experiment Setup

We conduct the experiments on an Ubuntu 20.04 machine with a 10-core 2.40 GHz Intel(R) Xeon(R) Silver 4210R CPU, 256G RAM, and 10G NVIDIA GeForce RTX 3080 GPU. All our experiments are based on the Pytorch framework.

#### 6.1.1. Data Collection

As shown in Table 2, we train a group of benign and malicious models on each of the three datasets CIFAR-10 [4], MNIST [21], and CelebA [22]. CIFAR-10 and MNIST both contain 60,000 images and their test sets both contain 10,000 images. CelebA contains 202,599 images and its test set consists of 19,962 images. In each group, we use three network architectures, ResNet [15], PreActResNet [23], and VGG16 [24], to train the benign and malicious models with 100 epochs. For each network architecture combined with each of the four regularization terms, we train ten instances, which are obtained using different hyper-parameters and initialized parameters. The coefficient of the regularization term is a hyper-parameter. Usually, the coefficient of $\Omega_{l_{1}}$ or $\Omega_{l_{2}}$ is small, such as 0.0001. We select the coefficients of $\Omega_{COR}$ and $\Omega_{SGN}$ such that the attacker can recover the training data by the correlated value and sign encoding attacks. For example, we use 8.0 as the coefficient of $\Omega_{COR}$ in ResNet trained on CIFAR-10. In addition, we train ten instances for each network architecture without any regularization terms. Overall, we train 20 malicious and 30 benign instances for each network architecture. There are 60 malicious and 90 benign models in each group, with 18 malicious and 27 benign models used for testing.

#### 6.1.2. Feature Extraction

**Figure 11:**
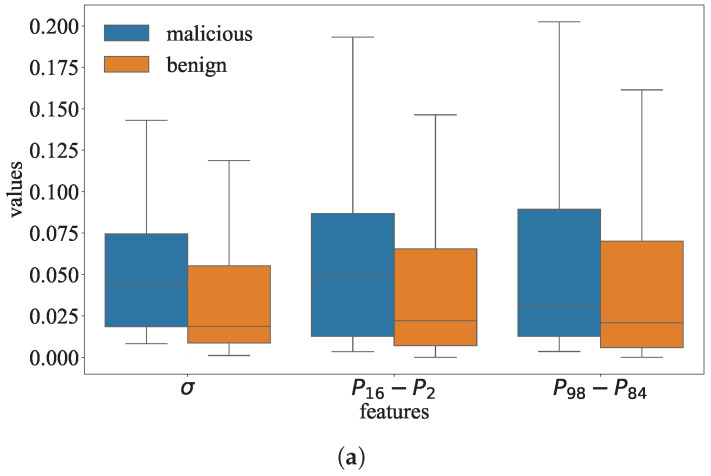

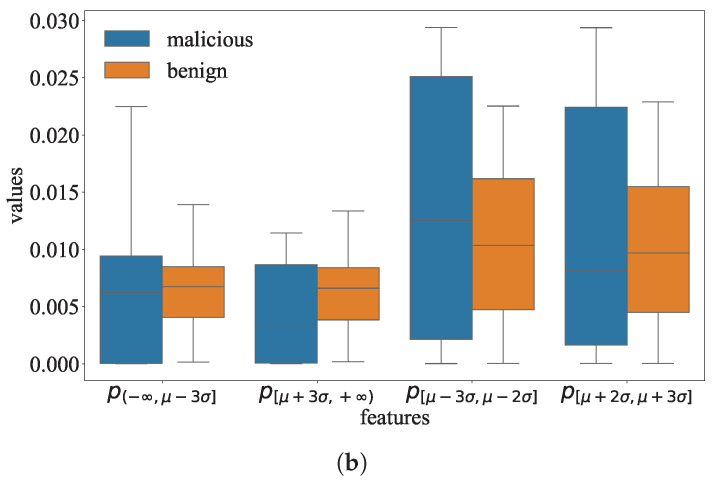
Comparison of the features extracted from the parameters of the malicious and benign models. (**a**) Features in Phenomenon 1. (**b**) Features in Phenomenon 2.

As described in Section 4.2, we use the standard deviation of the parameter distribution as a feature. Figure 11a shows that the malicious and benign models have notable differences in the inter-percentile ranges $P_{16}-P_{2}$ and $P_{98}-P_{84}$, so we choose $P_{16}-P_{2}$ and $P_{98}-P_{84}$ as the inter-percentile range features. As described in Section 4.2, we use $p_{\left(-\infty ,\mu -3\sigma \right]}$ and $p_{\left[\mu +3\sigma ,+\infty \right)}$ as two features to reflect the difference in the tail regions of the parameter distribution. In addition, we use $p_{\left[\mu -3\sigma ,\mu -2\sigma \right]}$ and $p_{\left[\mu +2\sigma ,\mu +3\sigma \right]}$ as two features to reflect the difference in the middle regions of the parameter distribution. As shown in Figure 11b, the malicious and benign models have notable differences in these four features. The Wilcoxon rank sum test is used to evaluate whether these features are significantly different between the malicious and benign models. The *p*-values of the Wilcoxon rank sum test are shown in Table 3. At a significance level of 0.05, almost all of the *p*-values for these features are below the significance level in all three datasets. Therefore, there is statistically significant evidence that these features are different between the malicious and benign models. Goalie extracts the above seven features for each of the first *k* training epochs of the instance. These features form the feature sequence of the instance. For each dataset, we obtain 150 sequences.

#### 6.1.3. Judgment

In our experiments, we use a single-layer BLSTM. The MLP contains two hidden layers using the Rectified Linear Unit (ReLU) as the activation function. Batch normalization is applied before each hidden layer. For each experimental group in Table 2, we use 70% malicious and benign sequences from all three network architectures as training data to train the BLSTM and MLP together, and the remaining 30% sequences as testing data.

#### 6.1.4. Evaluation Tasks

**Detection Effectiveness:** We use Goalie to detect the malicious models in the three experimental groups in Table 2. Goalie extracts parameter features from the model in the first *k* training epochs to detect whether the model is malicious or not, where *k* ranges from 5 to 30. The detection effectiveness of Goalie is evaluated in different numbers of training epochs. The metrics used are the accuracy, precision, recall, and $F_{1}$ of Goalie.

**Figure 12:**
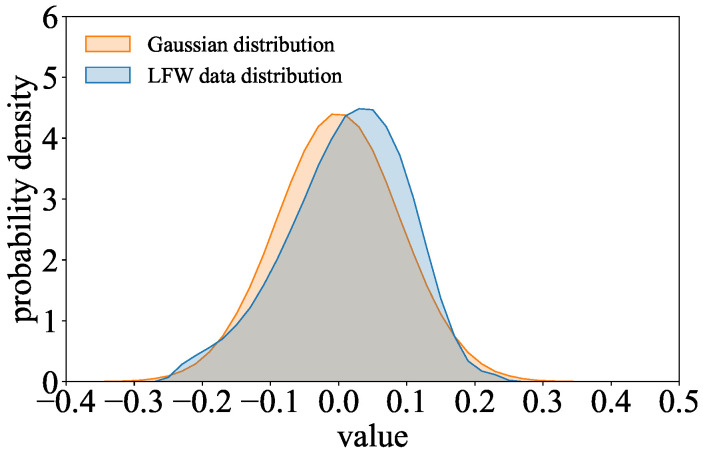
Comparison of the gray-scale value distribution of the LFW dataset and the Gaussian distribution.

**Detection Under Dataset Following Gaussian-Like Distribution:** As summarized in Section 5, when the training dataset follows a Gaussian-like distribution, the parameter distribution of the COR-model and $l_{2}$-model may be similar. We evaluate the effectiveness of Goalie in detecting models trained on a dataset that follows a Gaussian-like distribution. In the experiment, we use an image dataset whose pixel values follow a Gaussian-like distribution [25]. Specifically, we select the images of the person who appears more than 30 times in LFW [26] as the dataset, which can help the models to learn the relevant information about a person sufficiently. As shown in Figure 12, the gray-scale value distribution of LFW is similar to the Gaussian distribution. Other settings are the same as in the previous experiment.

**Detection Under Adversarial Attack:** An attacker can simultaneously add benign and malicious regularization terms to the model to confuse the detection. We evaluate the effectiveness of Goalie in detecting models with both malicious and benign regularization terms. There are four combinations of regularization terms used in the malicious models, namely $\Omega_{COR}$ and $\Omega_{l_{1}}$, $\Omega_{COR}$ and $\Omega_{l_{2}}$, $\Omega_{SGN}$ and $\Omega_{l_{1}}$, and $\Omega_{SGN}$ and $\Omega_{l_{2}}$. These malicious models are trained on the CIFAR-10 dataset by all three network architectures. We select the coefficients of the malicious and benign regularization terms such that the attacker can recover the training data through the correlated value and sign encoding attacks. For example, we use 32.0 and 0.0001 as the coefficients of $\Omega_{COR}$ and $\Omega_{l_{1}}$. The benign models used in this experiment are the benign models in group 1 of Table 2. Other settings are the same as in the previous experiment.

**Detection Efficiency:** We evaluate the detection efficiency of Goalie by computing the time for Goalie to detect a model. For each experimental group in Table 2, we compute the execution time forGoalie to detect the models when the models are trained from 5 to 30 epochs. We compare the time cost of Goalie in detecting models using the features extracted from the model in different training epochs to show the effect of the number of features on the efficiency of Goalie.

### 6.2. Experiment Results

**Figure 13:**
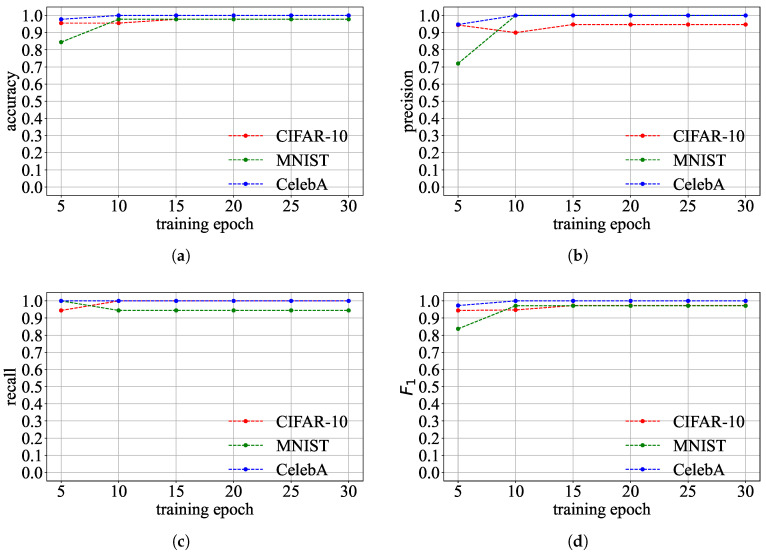
Detection effectiveness of Goalie. (**a**) Accuracy of Goalie in detecting benign and malicious models. (**b**) Precision of Goalie in detecting malicious models. (**c**) Recall of Goalie in detecting malicious models. (**d**) $F_{1}$ score of Goalie in detecting malicious models.

**Detection Effectiveness:** Figure 13 shows Goalie’s detection effectiveness when using the features extracted from different numbers of training epochs. The detection effectiveness of Goalie on the CIFAR-10 and CelebA datasets is always greater than 0.9, regardless of the number of training epochs used to extract parameter features. The accuracy, precision, and $F_{1}$ score of Goalie are less than 0.9 on the MNIST dataset when Goalie only uses the parameter features extracted from the first five epochs of the model. However, Goalie’s accuracy, precision, and $F_{1}$ score improve as the number of training epochs increases. This means that using more training epochs can make Goalie detect benign and malicious models more accurately. Although the recall of Goalie on the MNIST dataset in detecting malicious models decreases as the number of training epochs increases, the recall remains relatively high and exceeds 0.9. This indicates that Goalie can detect most malicious models. The detection effectiveness of Goalie when using the features extracted from the first 30 epochs of the model is shown in Table 4. The accuracy, precision, recall, and $F_{1}$ score of Goalie are above 0.9 on the three datasets.

**Figure 14:**
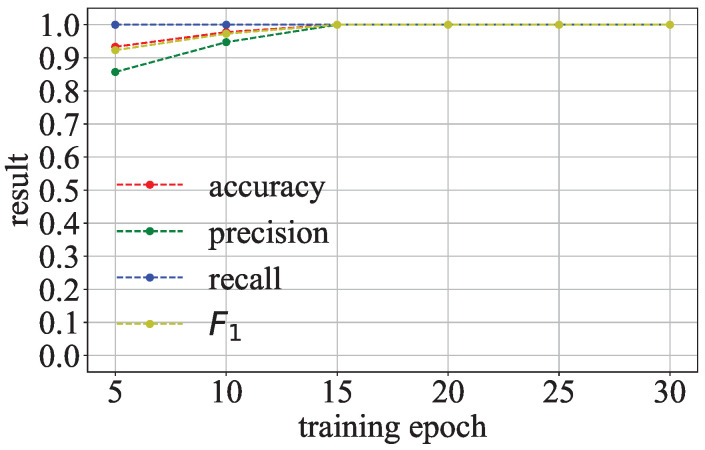
Effectiveness of Goalie in detecting models trained on the dataset whose distribution is close to a Gaussian distribution.

**Detection Under Dataset Following Gaussian-Like Distribution:** Figure 14 shows the effectiveness of Goalie in detecting the models trained on LFW when using the features extracted from different numbers of training epochs. Goalie’s accuracy, precision, recall, and $F_{1}$ score are close to or above 0.9 when Goalie only uses the parameter features extracted from the first five epochs of the model. As the number of training epochs increases, these metrics improve and eventually reach 1.0. This demonstrates that the features Goalie extracted based on the two discrepancy phenomena we found can be used to distinguish malicious models from benign models, even if the dataset follows a Gaussian-like distribution.

**Figure 15:**
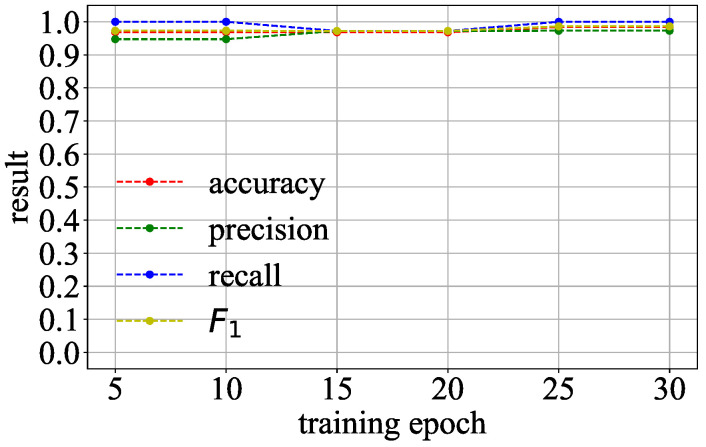
Detection effectiveness of Goalie in detecting models with malicious and benign regularization terms.

**Detection Under Adversarial Attack:** Figure 15 shows the detection effectiveness of Goalie in detecting models with malicious and benign regularization terms when using the features extracted from different numbers of training epochs. As the number of training epochs increases, Goalie’s precision, recall, and $F_{1}$ score significantly improve. The detection effectiveness of Goalie in detecting models with malicious and benign regularization terms when using the features extracted from the first 30 epochs of the model is shown in Table 5. The recall of Goalie can reach 1.0, and the precision of Goalie is 0.9. The $F_{1}$ score of Goalie is 0.95. This means that Goalie can also detect models with malicious and benign regularization terms.

**Figure 16:**
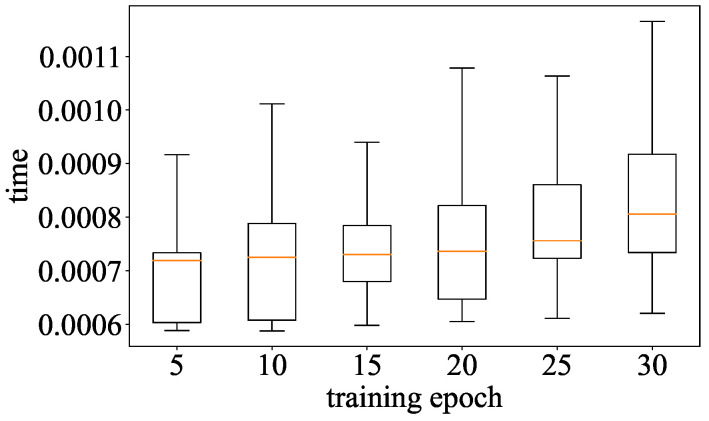
Detection efficiency of Goalie.

**Detection Efficiency:** The time for Goalie to detect a model for different training epochs is shown in Figure 16. When only using the parameter features of the model from the first five training epochs, the detection time is about 0.7 ms. The detection time of Goalie increases by only about 0.2 ms when the number of training epochs used to extract parameter features increases from 5 to 30. The maximum detection time is only about 1.1 ms when using the parameter features extracted from the model’s first 30 training epochs. This means that Goalie has a high detection efficiency.

## 7. Related Work

In this section, we describe the recent literature on defense and attack methods for the training data security of machine learning models.

### 7.1. Defenses

**Data Modification Methods:** Defenders can protect the training data of machine learning models by adding perturbations to these data [7,8]. Zhang, He, and Lee [7] use an obfuscate function before training a model to add random noise to existing samples or augment the dataset with new samples. This method can eliminate the leakage of properties of both individual samples and groups of samples. Defenders can also use the Homomorphic Encryption technique to achieve encrypted computation of private data and prevent data leakage [9,10,27,28]. Zhang, Yang, and Chen [9] propose a fully homomorphic encryption scheme to use encrypted data to train the deep learning models. Rahulamathavan et al. [10] use the Paillier encryption system to design an SVM decision function that can compute encrypted data. CryptoQFL [28] is a Quantum Federated Learning framework that allows distributed Quantum Neural Network training on encrypted data. Adding perturbations to the training data can reduce the quality of the data recovered by the attackers. However, this method also degrades the performance of the model. Encrypting the training data prevents the attacker from recovering the original data. However, this method reduces the computational efficiency of the model. Our method protects the data by detecting malicious models, which has a high efficiency.

**Model Modification Methods:** Defenders can modify the model’s gradient [5], parameters [6], or output results [29,30,31] to prevent attackers from capturing training data through the model. Abadi et al. [5] add noise to the gradient of the model parameters during the model training process to make the model preserve differential privacy. Golatkar, Achille, and Soatto [6] add noise to the model parameters to make the model forget the information of a specific subset in the training data. Jia et al. [30] add noise to the probabilistic output of the model with a certain probability to make it difficult for the attacker to determine if the input of the model is in the training data or not through the membership inference attack. Wang et al. [31] perform perturbation injection on a graph by adding noise to the intermediate output of graph neural networks to defend against white-box membership inference attacks. Defenders can also modify the training algorithm of the model so that the model stores less information about its training data [32,33,34,35]. Cao and Yang [32] use some summations to implement model training, so that only a small number of terms need to be recalculated if we want to forget a training sample. Salem et al. [33] use model stacking to prevent the model from overfitting the training data by combining multiple models trained on different subsets of the training data to construct the final model. Gao et al. [35] use the statistical information of the data to train models and apply knowledge distillation to isolate the relationship between gradients and training data to ensure that the training data cannot be recovered. Although modifying the model’s gradients or parameters can defend against the attack used to encode data in the model parameters, these methods all add noise to the model and affect the benign model when defending against attacks. Our method detects the models without modifying them, which can guarantee the performance of the benign models.

### 7.2. Attacks

**Model Inversion Attack:** Attackers recover a model’s training data through the model’s prediction results. If attackers can obtain the model, they can also use the parameters or intermediate results of the model to recover higher-quality training data of that model [3,20,36,37,38,39,40,41,42,43,44,45]. Song, Ristenpart, and Shmatikov [3] propose three ways to encode training data in the model, including encoding the training data in the lowest significant bits of the model parameters, encoding the training data in the model parameters, and encoding the training data in the labels of the additional malicious training data. Zhang et al. [39] propose a generative model inversion attack. They exploit partial public information to learn a prior distribution of the training data through generative adversarial networks (GANs) [46] and use this prior distribution to guide the model inversion process. Zhu and Han [40] perform a model inversion attack through the gradients of a model in Federated Learning. They obtain the gradients from the model by giving the model some randomly generated pairs of inputs and labels, and then optimize the generated inputs and labels to minimize the distance between the gradients and the real gradients from the Federated Learning process. Carlini et al. [42] demonstrate that large language models memorize and leak individual training data. They guide a large language model to output its training data by giving it a carefully designed prompt. PCEA [20] leverages the linear combinations of parameters to remember the training data during model training. Following the insight that the aggregate gradient from a fully connected (FC) layer is a linear combination of its inputs, CPA [45] adapts independent component analysis to recover private inputs for FC and convolutional networks.

**Membership Inference Attack:** Attackers provide a sample to a model to obtain the model’s prediction result, and then they can determine whether the sample is in the model’s training set based on the prediction result [33,47,48,49,50,51,52,53,54]. Shokri et al. [47] implement a membership inference attack by training multiple "shadow" models that simulate the target model. The training and testing set of "shadow" models are labeled as member and non-member to train an attack model to determine whether the input of the target model is in the training set based on the output of the target model. Nasr, Shokri, and Houmansadr [49] launched a white-box membership inference attack using the model’s gradient, hidden layer output, and objective function. They also designed an active membership inference attack in Federated Learning. Liu et al. [54] propose a new membership inference attack against Variational Auto-Encoders [55] and a GAN. They search for potential encodings of the target data to reconstruct the data, and then use the reconstruction error to determine whether the target data are in the training data of the generative network. TrajectoryMIA [56] represents the membership information by the loss trajectory evaluated on a sequence of intermediate models distilled from the target model at different distillation epochs, and builds an attack model to use the loss trajectory to infer membership.

## 8. Conclusions

We propose a method named Goalie that can defend against correlated value and sign encoding attacks by detecting and stopping the malicious models in these attacks. Goalie extracts some parameter distribution features from the models to distinguish the malicious models from the benign models. Our theoretical analysis demonstrates that the malicious and benign regularization terms make the model parameter distributions different, which confirms the validity of the features extracted by Goalie.

## Figures and Tables

**Table 1:** Features of model parameter distribution.

Phenomenon	Features
1	σ, $P_{b}-P_{a}$
2	$p_{\left[\mu -3\sigma ,\mu -2\sigma \right]}$, $p_{\left[\mu +2\sigma ,\mu +3\sigma \right]}$, $p_{\left(-\infty ,\mu -3\sigma \right]}$, $p_{\left[\mu +3\sigma ,+\infty \right)}$

**Table 2:** Data used in experimental groups.

Group	Dataset	Network Architecture	Malicious Sequences	Benign Sequences
1	CIFAR-10	ResNet	20	30
		PreActResNet	20	30
		VGG16	20	30
2	MNIST	ResNet	20	30
		PreActResNet	20	30
		VGG16	20	30
3	CelebA	ResNet	20	30
		PreActResNet	20	30
		VGG16	20	30

**Table 3:** The *p*-value of the Wilcoxon rank sum test between the features of the malicious and benign models in the 30th training epoch.

Dataset	σ	$P_{16}-P_{2}$	$P_{98}-P_{84}$	$p_{\left(-\infty ,\mu -3\sigma \right]}$	$p_{\left[\mu +3\sigma ,+\infty \right)}$	$p_{\left[\mu -3\sigma ,\mu -2\sigma \right]}$	$p_{\left[\mu +2\sigma ,\mu +3\sigma \right]}$
CIFAR-10	3.6 × 10^−4^	1.4 × 10^−4^	9.2 × 10^−5^	1.1 × 10^−3^	8.6 × 10^−4^	1.1 × 10^−4^	1.4 × 10^−3^
MNIST	4.1 × 10^−5^	2.1 × 10^−4^	1.5 × 10^−2^	4.2 × 10^−2^	3.5 × 10^−2^	4.8 × 10^−2^	6.3 × 10^−1^
CelebA	6.2 × 10^−5^	2.7 × 10^−3^	9.3 × 10^−4^	7.2 × 10^−3^	1.2 × 10^−2^	2.2 × 10^−2^	2.4 × 10^−2^

**Table 4:** Detection effectiveness of Goalie when using the features extracted from the first 30 epochs.

Dataset	Number of Detected Malicious Models	Number of Detected Benign Models	Accuracy	Precision	Recall	$F_{1}$ Score
CIFAR-10	18	26	0.98	0.95	1.0	0.97
MNIST	17	27	0.98	1.0	0.94	0.97
CelebA	18	27	1.0	1.0	1.0	1.0
LFW	18	27	1.0	1.0	1.0	1.0

**Table 5:** Effectiveness of Goalie in detecting models with malicious and benign regularization terms using features extracted from the first 30 epochs.

Regularization Terms	Precision	Recall	$F_{1}$ Score
$\Omega_{COR}$ & $\Omega_{l_{1}}$	0.90	1.0	0.95
$\Omega_{COR}$ & $\Omega_{l_{2}}$	0.90	1.0	0.95
$\Omega_{SGN}$ & $\Omega_{l_{1}}$	0.90	1.0	0.95
$\Omega_{SGN}$ & $\Omega_{l_{2}}$	0.90	1.0	0.95

## Data Availability

The associated datasets CIFAR-10, MNIST, CelebA and LFW used for the demonstration are publicly available. The link for CIFAR-10 is https://www.cs.toronto.edu/~kriz/cifar.html, accessed on 15 May 2024. The link for MNIST is https://www.kaggle.com/datasets/hojjatk/mnist-dataset, accessed on 15 May 2024. The link for CelebA is http://mmlab.ie.cuhk.edu.hk/projects/CelebA.html, accessed on 15 May 2024. The link for LFW is https://www.kaggle.com/datasets/atulanandjha/lfwpeople, accessed on 15 May 2024.

## References

[1] O’hara K. (2019). Data Trusts: Ethics, Architecture and Governance for Trustworthy Data Stewardship.

[2] Delacroix S., Montgomery J. (2020). From Research Data Ethics Principles to Practice: Data Trusts as a Governance Tool.

[3] Song C., Ristenpart T., Shmatikov V. Machine learning models that remember too much. Proceedings of the 2017 ACM SIGSAC Conference on Computer and Communications Security.

[4] Krizhevsky A. (2009). Learning Multiple Layers of Features from Tiny Images. Master’s Thesis.

[5] Abadi M., Chu A., Goodfellow I., McMahan H.B., Mironov I., Talwar K., Zhang L. Deep learning with differential privacy. Proceedings of the ACM SIGSAC Conference on Computer and Communications Security.

[6] Golatkar A., Achille A., Soatto S. Eternal sunshine of the spotless net: Selective forgetting in deep networks. Proceedings of the IEEE/CVF Conference on Computer Vision and Pattern Recognition.

[7] Zhang T., He Z., Lee R.B. (2018). Privacy-preserving machine learning through data obfuscation. arXiv.

[8] Jia J., Gong N.Z. Attriguard: A practical defense against attribute inference attacks via adversarial machine learning. Proceedings of the USENIX Security Symposium.

[9] Zhang Q., Yang L.T., Chen Z. (2016). Privacy Preserving Deep Computation Model on Cloud for Big Data Feature Learning. IEEE Trans. Comput..

[10] Rahulamathavan Y., Phan R.C.W., Veluru S., Cumanan K., Rajarajan M. (2013). Privacy-preserving multi-class support vector machine for outsourcing the data classification in cloud. IEEE Trans. Dependable Secur. Comput..

[11] Grm K., Štruc V., Artiges A., Caron M., Ekenel H.K. (2017). Strengths and weaknesses of deep learning models for face recognition against image degradations. IET Biom..

[12] Dodge S., Karam L. A study and comparison of human and deep learning recognition performance under visual distortions. Proceedings of the International Conference on Computer Communication and Networks (ICCCN).

[13] Nettleton D.F., Orriols-Puig A., Fornells A. (2010). A study of the effect of different types of noise on the precision of supervised learning techniques. Artif. Intell. Rev..

[14] Liu J., Lu Y.H., Koh C.K. (2010). Performance Analysis of Arithmetic Operations in Homomorphic Encryption.

[15] He K., Zhang X., Ren S., Sun J. Deep residual learning for image recognition. Proceedings of the IEEE Conference on Computer Vision and Pattern Recognition Workshops.

[16] Panaretos V.M., Zemel Y. (2019). Statistical Aspects of Wasserstein Distances. Annu. Rev. Statist. Appl..

[17] Han X., Zhang Z., Ding N., Gu Y., Liu X., Huo Y., Qiu J., Yao Y., Zhang A., Zhang L. (2021). Pre-trained models: Past, present and future. AI Open.

[18] Kreyszig E. (2011). Advanced Engineering Mathematics.

[19] Chernick M.R. (2011). The Essentials of Biostatistics for Physicians, Nurses, and Clinicians.

[20] Luo W., Zhang L., Han P., Liu C., Zhuang R. (2022). Taking Away Both Model and Data: Remember Training Data by Parameter Combinations. IEEE Trans. Emerg. Top. Comput. Intell..

[21] LeCun Y. (1998). The MNIST Database of Handwritten Digits. https://www.kaggle.com/datasets/hojjatk/mnist-dataset.

[22] Liu Z., Luo P., Wang X., Tang X. Deep learning face attributes in the wild. Proceedings of the IEEE International Conference on Computer Vision.

[23] He K., Zhang X., Ren S., Sun J. (2016). Identity mappings in deep residual networks. Lecture Notes in Computer Science.

[24] Simonyan K., Zisserman A. Very Deep Convolutional Networks for Large-Scale Image Recognition. Proceedings of the 3rd International Conference on Learning Representations, ICLR 2015.

[25] Xu L., Skoularidou M., Cuesta-Infante A., Veeramachaneni K. Modeling tabular data using conditional GAN. Proceedings of the Neural Information Processing Systems.

[26] Huang G.B., Ramesh M., Berg T., Learned-Miller E. (2007). Labeled Faces in the Wild: A Database for Studying Face Recognition in Unconstrained Environments.

[27] Phong L.T., Aono Y., Hayashi T., Wang L., Moriai S. (2018). Privacy-Preserving Deep Learning via Additively Homomorphic Encryption. IEEE Trans. Inf. Forensics Secur..

[28] Chu C., Jiang L., Chen F. CryptoQFL: Quantum Federated Learning on Encrypted Data. Proceedings of the 2023 IEEE International Conference on Quantum Computing and Engineering (QCE).

[29] Papernot N., Abadi M., Erlingsson Ú., Goodfellow I.J., Talwar K. Semi-supervised Knowledge Transfer for Deep Learning from Private Training Data. Proceedings of the 5th International Conference on Learning Representations, ICLR 2017.

[30] Jia J., Salem A., Backes M., Zhang Y., Gong N.Z. Memguard: Defending against black-box membership inference attacks via adversarial examples. Proceedings of the 2019 ACM Conference on Computer and Communications Security.

[31] Wang K., Wu J., Zhu T., Ren W., Hong Y. (2023). Defense against membership inference attack in graph neural networks through graph perturbation. Int. J. Inf. Sec..

[32] Cao Y., Yang J. Towards making systems forget with machine unlearning. Proceedings of the IEEE Symposium on Security and Privacy.

[33] Salem A., Zhang Y., Humbert M., Fritz M., Backes M. ML-Leaks: Model and Data Independent Membership Inference Attacks and Defenses on Machine Learning Models. Proceedings of the Network and Distributed System Security Symposium.

[34] Nasr M., Shokri R., Houmansadr A. Machine learning with membership privacy using adversarial regularization. Proceedings of the ACM Conference on Computer and Communications Security.

[35] Gao K., Zhu T., Ye D., Zhou W. (2024). Defending against gradient inversion attacks in federated learning via statistical machine unlearning. Knowl. Based Syst..

[36] Fredrikson M., Jha S., Ristenpart T. Model inversion attacks that exploit confidence information and basic countermeasures. Proceedings of the ACM Conference on Computer and Communications Security.

[37] Yang Z., Zhang J., Chang E.C., Liang Z. Neural network inversion in adversarial setting via background knowledge alignment. Proceedings of the ACM Conference on Computer and Communications Security.

[38] Salem A., Bhattacharya A., Backes M., Fritz M., Zhang Y. Updates-leak: Data set inference and reconstruction attacks in online learning. Proceedings of the USENIX Security Symposium.

[39] Zhang Y., Jia R., Pei H., Wang W., Li B., Song D. The secret revealer: Generative model-inversion attacks against deep neural networks. Proceedings of the IEEE Conference on Computer Vision and Pattern Recognition.

[40] Zhu L., Han S. (2020). Deep leakage from gradients. Lecture Notes in Computer Science.

[41] Hitaj B., Ateniese G., Perez-Cruz F. Deep models under the GAN: Information leakage from collaborative deep learning. Proceedings of the ACM SIGSAC Conference on Computer and Communications Security.

[42] Carlini N., Tramèr F., Wallace E., Jagielski M., Herbert-Voss A., Lee K., Roberts A., Brown T., Song D., Erlingsson Ú. Extracting Training Data from Large Language Models. Proceedings of the USENIX Security Symposium.

[43] Pan X., Zhang M., Ji S., Yang M. Privacy risks of general-purpose language models. Proceedings of the IEEE Symposium on Security and Privacy.

[44] Carlini N., Liu C., Erlingsson Ú., Kos J., Song D. The secret sharer: Evaluating and testing unintended memorization in neural networks. Proceedings of the USENIX Security Symposium.

[45] Kariyappa S., Guo C., Maeng K., Xiong W., Suh G.E., Qureshi M.K., Lee H.H.S. Cocktail party attack: Breaking aggregation-based privacy in federated learning using independent component analysis. Proceedings of the 40th International Conference on Machine Learning, ICML’23.

[46] Goodfellow I.J., Pouget-Abadie J., Mirza M., Xu B., Warde-Farley D., Ozair S., Courville A.C., Bengio Y. Generative Adversarial Nets. Proceedings of the Advances in Neural Information Processing Systems.

[47] Shokri R., Stronati M., Song C., Shmatikov V. Membership inference attacks against machine learning models. Proceedings of the IEEE Symposium on Security and Privacy.

[48] Song L., Shokri R., Mittal P. Privacy risks of securing machine learning models against adversarial examples. Proceedings of the ACM Conference on Computer and Communications Security.

[49] Nasr M., Shokri R., Houmansadr A. Comprehensive privacy analysis of deep learning: Passive and active white-box inference attacks against centralized and federated learning. Proceedings of the Symposium on Security and Privacy.

[50] Melis L., Song C., De Cristofaro E., Shmatikov V. Exploiting unintended feature leakage in collaborative learning. Proceedings of the Symposium on Security and Privacy.

[51] Leino K., Fredrikson M. Stolen memories: Leveraging model memorization for calibrated white-box membership inference. Proceedings of the USENIX Security Symposium.

[52] Sablayrolles A., Douze M., Schmid C., Ollivier Y., Jégou H. White-box vs black-box: Bayes optimal strategies for membership inference. Proceedings of the International Conference on Machine Learning, ICML, PMLR.

[53] Hayes J., Melis L., Danezis G., De Cristofaro E. (2019). LOGAN: Membership Inference Attacks Against Generative Models. Proc. Priv. Enhancing Technol..

[54] Liu K.S., Xiao C., Li B., Gao J. Performing co-membership attacks against deep generative models. Proceedings of the IEEE International Conference on Data Mining.

[55] Kingma D.P., Welling M. Auto-Encoding Variational Bayes. Proceedings of the 2nd International Conference on Learning Representations, ICLR 2014.

[56] Liu Y., Zhao Z., Backes M., Zhang Y. Membership Inference Attacks by Exploiting Loss Trajectory. Proceedings of the 2022 ACM SIGSAC Conference on Computer and Communications Security, CCS ’22.

